# Surface area-dependence of gas-particle interactions influences pulmonary and neuroinflammatory outcomes

**DOI:** 10.1186/s12989-016-0177-x

**Published:** 2016-12-01

**Authors:** Christina R. Tyler, Katherine E. Zychowski, Bethany N. Sanchez, Valeria Rivero, Selita Lucas, Guy Herbert, June Liu, Hammad Irshad, Jacob D. McDonald, Barry E. Bleske, Matthew J. Campen

**Affiliations:** 1 Department of Pharmaceutical Sciences, The University of New Mexico College of Pharmacy, Albuquerque, NM USA; 2 Lovelace Respiratory Research Institute, Albuquerque, NM USA; 3 Department of Pharmacy Practice & Administrative Sciences, The University of New Mexico, Albuquerque, NM USA

## Abstract

**Background:**

Deleterious consequences of exposure to traffic emissions may derive from interactions between carbonaceous particulate matter (PM) and gaseous components in a manner that is dependent on the surface area or complexity of the particles. To determine the validity of this hypothesis, we examined pulmonary and neurological inflammatory outcomes in C57BL/6 and apolipoprotein E knockout (ApoE^−/−^) male mice after acute and chronic exposure to vehicle engine-derived particulate matter, generated as ultrafine (UFP) and fine (FP) sizes, with additional exposures using UFP or FP combined with gaseous copollutants derived from fresh gasoline and diesel emissions, labeled as UFP + G and FP + G.

**Results:**

The UFP and UFP + G exposure groups resulted in the most profound pulmonary and neuroinflammatory effects. Phagocytosis of UFP + G particles via resident alveolar macrophages was substantial in both mouse strains, particularly after chronic exposure, with concurrent increased proinflammatory cytokine expression of CXCL1 and TNFα in the bronchial lavage fluid. In the acute exposure paradigm, only UFP and UFP + G induced significant changes in pulmonary inflammation and only in the ApoE^−/−^ animals. Similarly, acute exposure to UFP and UFP + G increased the expression of several cytokines in the hippocampus of ApoE^−/−^ mice including *Il-1β*, *IL-6*, *Tgf-β* and *Tnf-α* and in the hippocampus of C57BL/6 mice including *Ccl5*, *Cxcl1*, *Il-1β*, and *Tnf-α*. Interestingly, *Il-6* and *Tgf-β* expression were decreased in the C57BL/6 hippocampus after acute exposure. Chronic exposure to UFP + G increased expression of *Ccl5, Cxcl1, Il-6*, and *Tgf-β* in the ApoE^−/−^ hippocampus, but this effect was minimal in the C57BL/6 mice, suggesting compensatory mechanisms to manage neuroinflammation in this strain.

**Conclusions:**

Inflammatory responses the lung and brain were most substantial in ApoE^−/−^ animals exposed to UFP + G, suggesting that the surface area-dependent interaction of gases and particles is an important determinant of toxic responses. As such, freshly generated UFP, in the presence of combustion-derived gas phase pollutants, may be a greater health hazard than would be predicted from PM concentration, alone, lending support for epidemiological findings of adverse neurological outcomes associated with roadway proximity.

**Electronic supplementary material:**

The online version of this article (doi:10.1186/s12989-016-0177-x) contains supplementary material, which is available to authorized users.

## Background

Epidemiological studies have identified that traffic emissions, or near-roadway exposures, are often associated with greater risk for cardiopulmonary and neurological morbidity than are other metrics of air pollution [1, 2]. While concentrations of exhaust components are highest at the source and become dilute with transport away from the roadway, there is also the likelihood that freshly generated particulate matter (PM) may have a greater toxicity than aged PM. Findings from recent toxicological studies suggest that an interaction may occur between particulate and gaseous components of vehicle exhausts that potentiates cardiopulmonary toxicity, although it was unclear if this effect was due to the additive toxicity of the two components or if PM toxicity was modified by the presence of adsorbed species [3]. Recent innovations in diesel exhaust reduction technology effectively lower PM emissions from vehicles by filtration, but gaseous components may still be as high or higher. These components can then interact with background PM or resuspended road dust during operation. Thus, improved understanding of gas-particle interactions is important for effective protection of human health.

The cardiovascular effects of vehicle engine-derived pollutants have been established in the literature, especially as they relate to diesel emissions [4–8] and, to a lesser extent, gasoline engine emissions [9]. More recently, however, compelling studies have noted a correlation between PM exposure and the onset of neurodegenerative disorders, such as Alzheimer’s disease (AD) [10]. Recent in vivo and in vitro studies have reported exposure to particulate matter induces adverse neurological outcomes, including neuroinflammation assessed by oxidative stress and cytokine production, associated with impaired cognitive function and neuropathology reminiscent of neurodegenerative disorders including AD and Parkinson’s disease [11–15]. The mechanism by which this toxicity in the brain occurs is still unknown; however, neuroinflammation, particularly priming of the brain’s resident immune cells, microglia, resulting in both detrimental and protective functions under pathological conditions, as measured by cytokine production, may underlie several cognitive and neurodegenerative disorders [16]. Additionally, chronic systemic inflammation resulting from cardiovascular disease (CVD) may be associated with an increased risk for developing neuroinflammation potentially leading to neurodegenerative disorders [17].

Inhaled particulate matter induces cardiovascular and respiratory inflammation [18–22]. Interactions between combustion-source particulate matter and associated gaseous components may potentiate toxicity, resulting in greater systemic inflammation and, potentially, neuroinflammation, thus increasing the risk for development of neurodegenerative disorders, like AD. We have previously demonstrated interactions between gaseous and particulate components in driving cardiovascular effects [3, 23]; however, it was unclear whether such interactions were influenced more by particulate morphology and surface area or chemical composition. In the present study, we hypothesize that smaller PM, with a higher surface area per mass, will have a greater interaction with gaseous co-pollutants and lead to exacerbated pulmonary and systemic toxicity. To assess this, we developed a complex exposure paradigm that allows for the vapor phase of mixed engine emissions to be combined with carbonaceous PM distinctly in ultrafine particle (UFP) or fine particle (FP) modes, which were then used for exposures in a sensitive model of vascular disease, the apolipoprotein E-deficient mouse (ApoE^−/−^). To our knowledge, this is the first report to provide toxicological evidence demonstrating pulmonary and neuroinflammatory outcomes that were determined by PM surface area and presumably physicochemical interactions with adsorptive gaseous species.

## Methods

### Development of atmosphere combinations

**Fig. 1:**
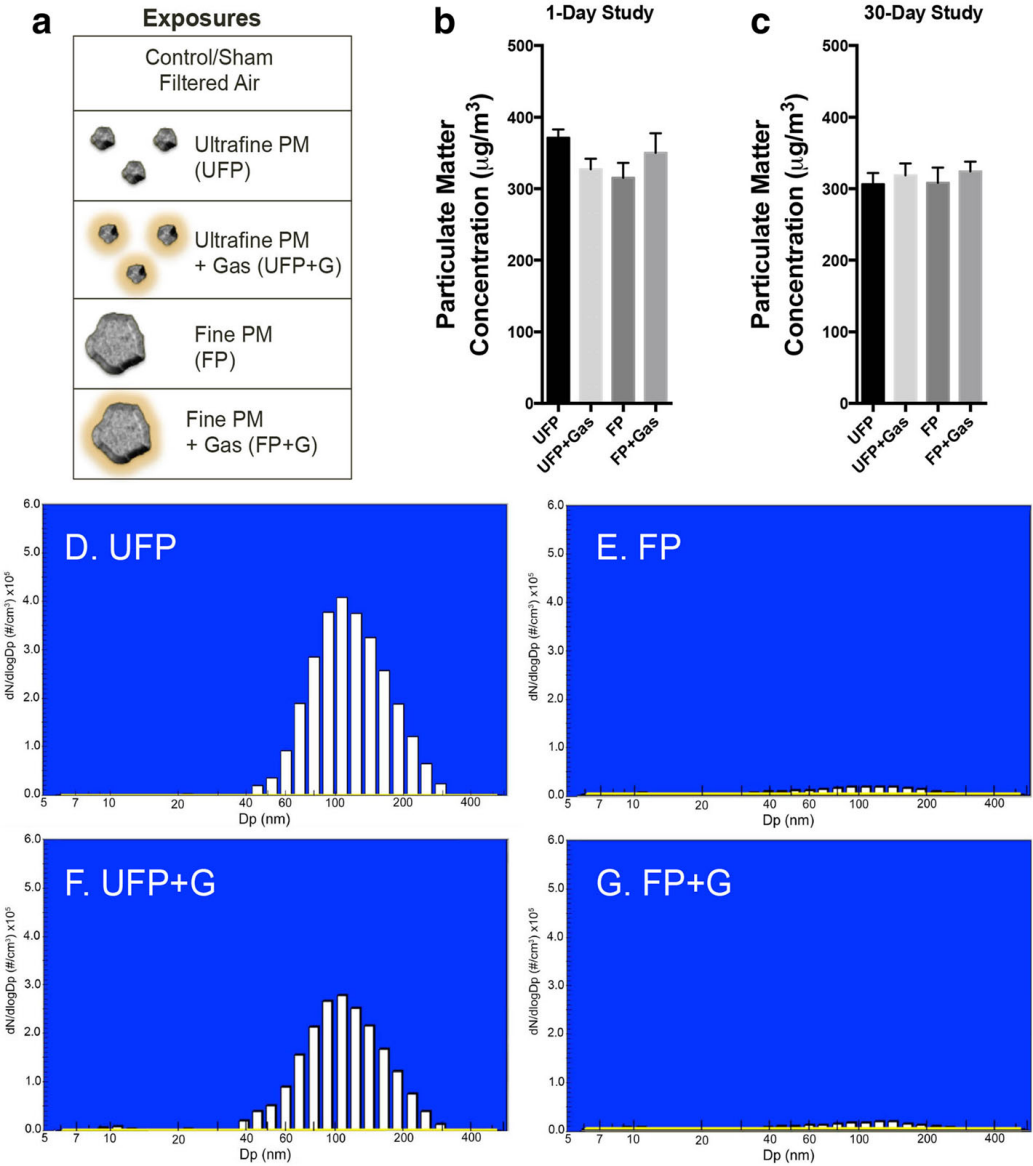
Atmospheric characterization. (**a**) Visual representation of each exposure group demonstrating the relevant size and complexity of ultrafine particulate matter (UFP), ultrafine particulate matter combined with vehicular gases (UFP + G), fine particulate matter (FP), and fine particulate matter combined with gases (FP + G). The particulate matter target concentration (300 μg/m^3^) was reached for each exposure group in both the (**b**) 1-day (acute) and (**c**) 30-day (chronic) studies. The distribution of the (**d**) ultrafine particle (UFP) group is in the nanometer range, similar to the distribution of the (**f**) ultrafine particles recombined with vehicular gases (UFP + G) group. Characterization of the (**e**) fine particle (FP) and (**g**) fine particles recombined with vehicular gases (FP + G) demonstrate that these exposures do not contain detectable PM in the nanometer ultrafine range suggesting larger particulate matter size

The exposure atmospheres are described in Fig. 1a, which included UFP, UFP with gaseous copollutants (UFP + G), FP, and FP with gaseous copollutants (FP + G). These core atmospheres were derived from mixed vehicle exhaust (MVE), which consisted of a combination of both diesel engine exhausts (DEE) and gasoline engine exhausts (GEE), as previously described [3, 24]. Target exposure mass concentration was 300 μg PM/m^3^ for all exposure groups. A description of the aerosol generation technique for each of the individual atmospheric components or mixtures is described below. Exhausts from a gasoline engine (GEE) and a diesel engine (DEE) were generated and characterized as previously reported. The exhausts were extracted via eduction pumps into a 2 m^3^ mixing chamber where they were combined and subsequently routed to one of several separate inhalation chambers.

#### Generation of the base MVE atmosphere

MVE is a model of combined DEE and GEE designed to reflect natural mixtures in near-roadway conditions and thus was used as the basis for the UFP and FP permutations. Specifically, target levels for PM and gases were derived from previous work with this fresh combustion mixture, and more detailed methods and schematics of the system are published elsewhere [3, 23]. DEE was produced from a single-cylinder, 5500-watt, Yanmar diesel-engine generator using a combination of heavy sulfur fuel and Number 2 Diesel Certification Fuel (Phillips Chemical Company) and 40 weight motor oil (Rotella T, Shell) as previously described [25]. Electrical current was pulled from the engine to provide a constant load (90%) during operation. The desired concentrations were attained by diluting the direct exhaust with filtered air. The air used for filtered air control exposures and also for dilution of engine emissions was pre-treated by passage through a carbon-impregnated filter to remove volatile organics and through a HEPA filter to remove PM. GEE was generated as previously described [26], with the notable exception that one engine was used during a 6 h exposure period instead of two engines. In brief, exhaust was generated from a 1996 General Motors 4.3 L V6 gasoline engine equipped with a stock exhaust system (including muffler and catalyst). The engine was connected to an eddy current dynamometer (Model Alpha 240, Zöllner, Kiel, West Germany) linked to a dynamometer interface (Type DTC-1, Dyne Systems Co., LLC, Germantown, WI) that was controlled by a custom software program (Cell Assistant, Dyne Systems Co.). The engine was fueled with gasoline obtained from a local station in Albuquerque, NM. Crankcase oil (10 W-30, Pennzoil Products Company, Houston, TX) and oil filter (Duraguard PF52, AC Delco, Detroit, MI) were changed every 122 h (equivalent to 3000 miles) of engine operation.

The MVE was then created by combining a dilution of GEE (diluted approximately 10:1 with filtered air) with a similar dilution of DEE to achieve a target of 300 μg PM/m^3^. Relative PM contributions to the MVE atmosphere reflected a ratio of GEE to DEE of approximately 1:5; however, GEE accounted for the majority of carbon monoxide and non-methane volatile organic compounds [3, 24]

#### Ultrafine particulate with gases (UFP + G) and ultrafine particulate without gases (UFP)

UFP + G atmosphere was generated using the MVE directly from a 1-m^3^ mixing chamber containing DEE and GEE. UFP atmosphere was generated by passing the MVE from 1-m^3^ mixing chamber through a Harvard denuder [23]. This denuder is a parallel plate opposite flow diffusion denuder with gaseous removal efficiency of 80–90%.

#### Fine Particulate with gases (FP + G) and fine particulate without gases (FP)

FP + G atmosphere was generated using the deposits from the exhaust line of the diesel engine. The deposits were packed in the delivery cup of a Wright Dust Feeder (WDF; CH Technologies, USA) and delivered to the inhalation exposure chamber using HEPA-filtered compressed air. The output from the WDF was passed through a cyclone with a cut-point of 2.5 μm (URG, Inc., Chapel Hill, NC) to remove large particulates from the aerosol. HEPA filtered gases from 1 m^3^ mixing chambers containing MVE were pumped into the exposure chamber. FP without gases atmosphere was generated using a similar technique as described above for FP + G; no HEPA filtered MVE gases were provided in the exposure chamber.

### Atmosphere monitoring and characterization

Test atmosphere characterization was conducted as previously described [25–27]. In brief, sample collection strategies were developed to capture and measure gas, semivolatile, and particle phases for a broad spectrum of chemical classes. Gases were analyzed by chemiluminescence (NOx) and infrared spectroscopy (CO). Particulate mass concentration was measured via gravimetrical analysis on 47-mm glass fiber filters (GE Whatman, Pittsburgh, PA). Particle size distribution was measured with a Fast Mobility Particle Sizer (FMPS, TSI, St. Paul, MN) for the ~10–500 nm size range and an Aerodynamic Particle Sizer (TSI, St Paul, MN) to measure the 0.5–20 μm size range.

### Whole-body inhalation exposures to atmospheres

C57BL/6 and ApoE^−/−^ male mice obtained from the Jackson Laboratory aged 6–8 weeks were pair-housed and maintained in controlled environment (30–60% relative humidity, 20–24 °C) on a 12-h light:dark cycle with *ad libitum* access to water and standard chow (2016C Harlan Global Certified Rodent Chow for C57BL/6 mice). ApoE^−/−^ mice were fed high-fat chow (TD.88137 Harlan, 21% fat, 0.2% cholesterol) for the entire period of study to model previously demonstrated vulnerability to vascular inflammatory conditions [5, 28]. Exposure chambers were also monitored for temperature and humidity to ensure adherence to AAALAC standards. Animals were randomized into the following groups*:* Filtered air (FA, control), UFP, UFP + G, FP, FP + G, using 8 mice per strain per group; in one group (ApoE^−/−^ mice in the FP + G group), four mice had to be excluded from post-exposure biological analyses for pre-existing health reasons. Animals were placed in 2-m^3^ whole-body rodent inhalation chambers (Lab Products, Inc., Maywood, NJ) and chow was removed during the daily exposures. Animals received either one 6-h whole body inhalation exposure (acute exposure) or 30 days of 6-h whole body inhalation exposure (chronic exposure) to 300 μg/m^3^ of the atmospheres (described above). All procedures were approved by the Lovelace Respiratory Research Institute’s Animal Care and Use Committee and conform to the Guide for the Care and Use of Laboratory Animals published by the US National Institutes of Health (NIH Publication No. 85–23, revised 1996). Animal body weights were monitored weekly throughout the study. All mice were fasted overnight prior to necropsy. Animals were euthanized via intraperitoneal injection of barbiturate-based sedative (Euthasol™). The following tissues were rapidly dissected, placed in liquid nitrogen, and stored at −80 °C until use: cerebrum (meninges, brain stem, cerebellum, OB removed), lung lobes, whole aorta (collected from ascending aorta before abdominal bifurcation), liver, heart (upper), heart (lower), and abdominal adipose tissue.

### Bronchoalveolar lavage (BALF) collection

BALF was collected via whole lung lavage with 800 μl Dulbecco’s PBS. After centrifugation, BALF supernatant was snap frozen in liquid nitrogen and stored at −80 °C until use. An aliquot of BALF supernatant was assayed for lactate dehydrogenase (LDH) and total protein as previously described [29].

BALF cells were used to prepare Cytospin slides for cell differential counting and histological scoring of particulate uptake. After fixation, the slides were stained with a modified Wright-Giemsa method, and subjected to qualitative scoring by a blind observer using the following scoring system under light microscope: 0, no particles; 1, scattered cells with intra-cellular particles; 3, slightly increased numbers of cells with particles, may not be easily detected on casual examination; 5, number of cells with particles is substantially greater than normal and can be easily noted upon examination; 7, the abundance of cells with particles is marked, and the intra-cellular particles are prominent or have severe cytosolic characters. The representative cell images were taken with an Olympus CCD camera through an oil immersion objective (×100).

### Cytokine analysis in BALF

Cytokine protein concentration was assessed in BALF using the V-Plex Proinflammatory Panel 1 Mouse Kit (Meso Scale Discovery, Gaithersburg, MD, USA) according to the manufacturer’s instructions. Briefly, plates were pre-coated with a capture antibody for the following cytokines: IFN-γ (interferon gamma), IL-10 (interleukin 10), IL-12p70 (interleukin 12 active heterodimer), IL-1β (interleukin 1 beta), IL-2 (interleukin 2), IL-4 (interleukin 4), IL-5 (interleukin 5), IL-6 (interleukin 6), KC/GRO (CXCL1, chemokine C-X-C motif ligand 1), and TNF-α (tumor necrosis factor alpha). BALF (50 μl/well) was added and allowed to react for 2 h at RT. Plates were washed 3× with buffer containing 1X PBS and 0.05% Tween 20. Detection antibody was added to each well and allowed to react for 1 h at RT. Plates were washed as previously noted and Read Buffer was added to each well. Plates were analyzed on MSD QuickPlex SQ 120 instrument (MSD, AI0AA-0); Discovery Workbench (v. 4.0) software calculated cytokine concentrations using a linear regression analysis of the standard curve.

### Transcription profiling in brain

#### Tissue preparation

The hippocampus and rest of cortex were dissected from the stored brains, placed in RNAlater (LifeTechnologies, AM7020), and immediately homogenized for 3 consecutive rounds using MagNA Lyzer beads (Roche, 03358941001) and homogenizer set at 6000 rpm for 60 s increments.

#### RNA isolation and qPCR

Total RNA was isolated from homogenized hippocampal tissue using the RNeasy Mini Kit (Qiagen, 74104). One microgram of high quality (260/280 = ~2.0 and 260/230 = ~1.0) RNA was converted cDNA using Applied Biosystem’s High Capacity cDNA Reverse Transcription Kit (LifeTechnologies, 4368814). Primer validation was performed for all TaqMan primers (LifeTechnologies) used in this study; genes of interest were matched with housekeeping genes of the same efficiency. Quantitative PCR was performed with 25 ng cDNA from hippocampal tissue; qPCR conditions were as follows: 2 min @ 50 °C, 10 min @ 95 °C, and 45 cycles of 15 s @ 95 °C and 60 s @ 60 °C. No template controls and RT- controls for each gene and each sample were used for each qPCR plate. Genes of interest (GOI) include: *Ccl2* (C-C motif chemokine ligand 2; Mm00441242_m1), *Ccl5* (C-C motif chemokine ligand 5; Mm01302427_m1), *Cxcl1* (chemokine C-X-C motif ligand 1; Mm04207460_m1), *Et-1* (endothelin 1; Mm00438656_m1), *Il-6* (interleukin 6; Mm00446190_m1), *Il-1β* (interleukin 1 beta; Mm00434228_m1), *Tgf-β* (transforming growth factor beta 1; Mm01178820_m1), *Tnf-α* (tumor necrosis factor alpha; Mm00443258_m1), *Vcam-1* (vascular cell adhesion molecule 1; Mm01320970_m1). Housekeeping genes (HKG) included *B2m* (beta-2-microglobulin; Mm00437762_m1), *Hprt* (hypoxanthine phosphoribosyltransferase Mm03024075_m1), and *Tbp* (TATA-box binding protein; Mm01277042_m1).

#### qPCR analysis

All data was run in duplicate; only samples with threshold cycle values (CT) under 35 were used for analysis. Average GOI CT values were normalized to average HKG CT values for associated HKGs. Subsequent ΔCT values were assessed using the comparative CT method (ΔΔCT), and results are expressed as fold change.

### Statistical analysis

One-way ANOVA (SPSS, v.19) with post-hoc analysis was performed as needed and corrected for multiple comparisons with Bonferroni, *n* = 6–8 per group for all analysis, except where indicated in the results section. Statistical significance was set at 95% confidence. Quantification of accumulation of particulate matter within resident alveolar macrophages was analyzed using the Kruskal-Wallis test for nonparametrics for both exposure paradigms in both strains of animals as each distribution failed the normality assumption of the one-way ANOVA.

## Results

### Characterization of inhalation exposure atmospheres

Concentrations of particulate matter, carbon monoxide, and nitrogen oxide gases for each of the test atmospheres during the acute and chronic exposures are provided in Table 1 and Fig. 1b, c. Extensive characterization of gases and particulate matter for this exposure paradigm is provided in previous reports [3, 23]. PM distribution in the ultrafine range is shown in Fig. 1d–g, with UFP atmospheres exhibiting comparable distributions (UFP mmad = 147.1 ± 1.3 nm; UFP + G mmad = 142.1 ± 1.3 nm), while FP atmospheres were largely devoid of detectable PM in the ultrafine range, despite close matching of the overall mass concentrations. Fine PM was approximately 10 times larger, within the low micron range; typical number median aerodynamic diameter (NMAD) and mass median aerodynamic diameter (MMAD) for FP and FP + G atmospheres, as measured by an Aerodynamic Particle Sizer, ranged from 1.5 to 3.0 μm ± 1.3–1.6 μm (geometric standard deviation).

**Table 1:** Test atmosphere concentrations of particulate matter (PM), CO, and NO_x_

Atmosphere	Exposure	PM (μg/m^3^)	NOx (ppm)	CO (ppm)
UFP	Acute (1 day)	371.3 ± 15.6	7.84 ± 4.14	16.77 ± 1.10
UFP + G	Acute (1 day)	327.3 ± 35.6	33.11 ± 5.35	27.93 ± 4.05
FP	Acute (1 day)	315.3 ± 50.7	0.10 ± 0.00^a^	11.60 ± 0.00^a^
FP + G	Acute (1 day)	350.3 ± 47.4	20.17 ± 23.26	73.85 ± 107.76
UFP	Chronic (30 days)	306.3 ± 86.2	4.24 ± 2.80	33.53 ± 40.66
UFP + G	Chronic (30 days)	324.2 ± 75.7	20.80 ± 11.51	107.5 ± 126.5
FP	Chronic (30 days)	308.4 ± 116.3	0.88 ± 2.17	22.03 ± 24.66
FP + G	Chronic (30 days)	318.7 ± 91.7	12.31 ± 10.27	49.36 ± 56.46

^a^Only one measurement was captured for gas concentrations in the FP chamber per day

### Body weight changes, pulmonary inflammation and macrophage uptake of PM

**Fig. 2:**
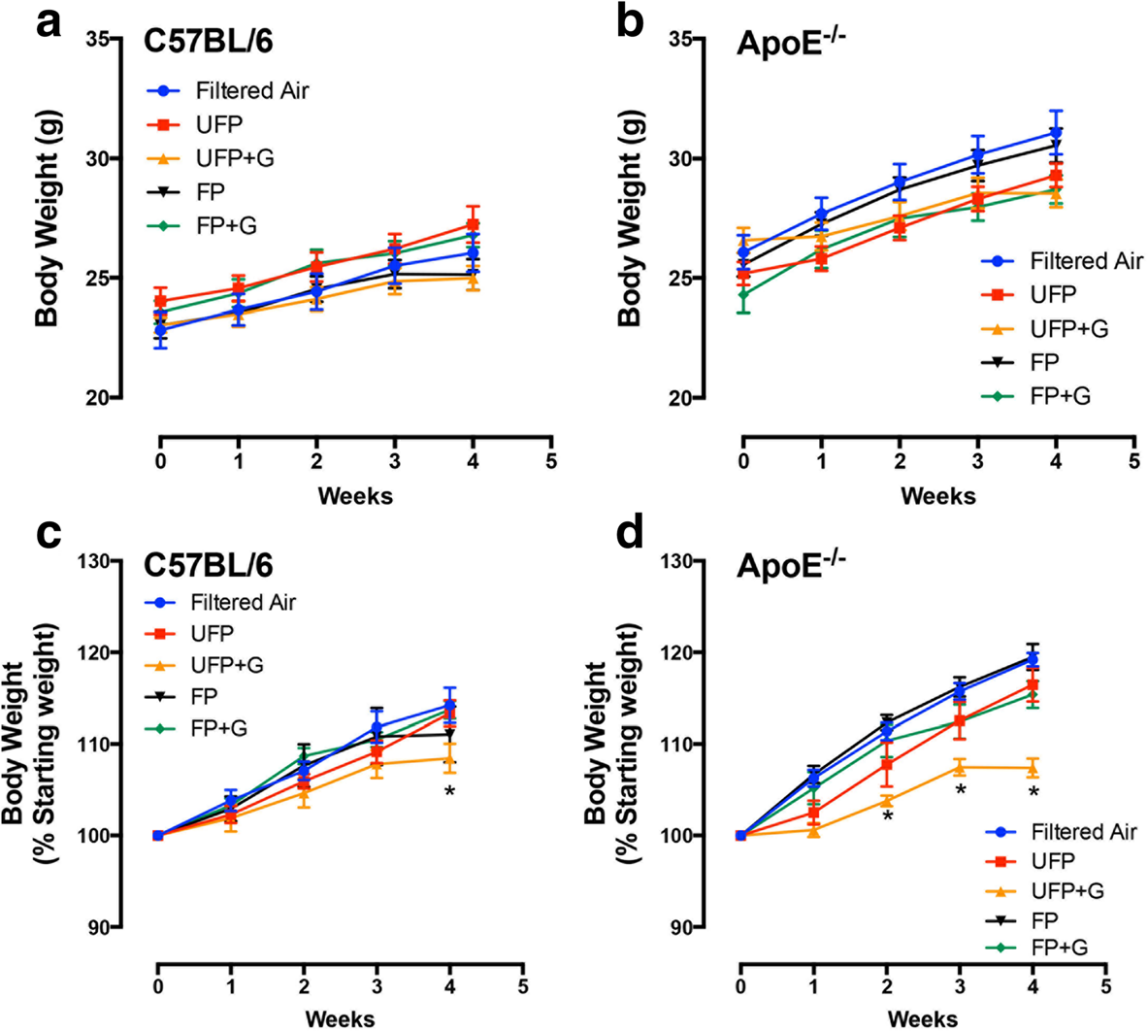
Assessment of body weights in C57BL/6 and ApoE^−/−^ mice. Body weight was determined weekly throughout the chronic exposure (30-day) paradigm. Body weight increased throughout the chronic exposure for (**a**) C57BL/6 male mice and (**b**) ApoE^−/−^ male mice, aged 6–8 weeks. Normalization of body weight to initial weight reveals significant reduction in growth due to UFP + G exposure in (**c**) C57BL/6 male mice at week four and in (**d**) ApoE^−/−^ male mice starting at week two. *n* = 7–8 per group, *p < .05; **p < .01; ***p < .001

**Fig. 3:**
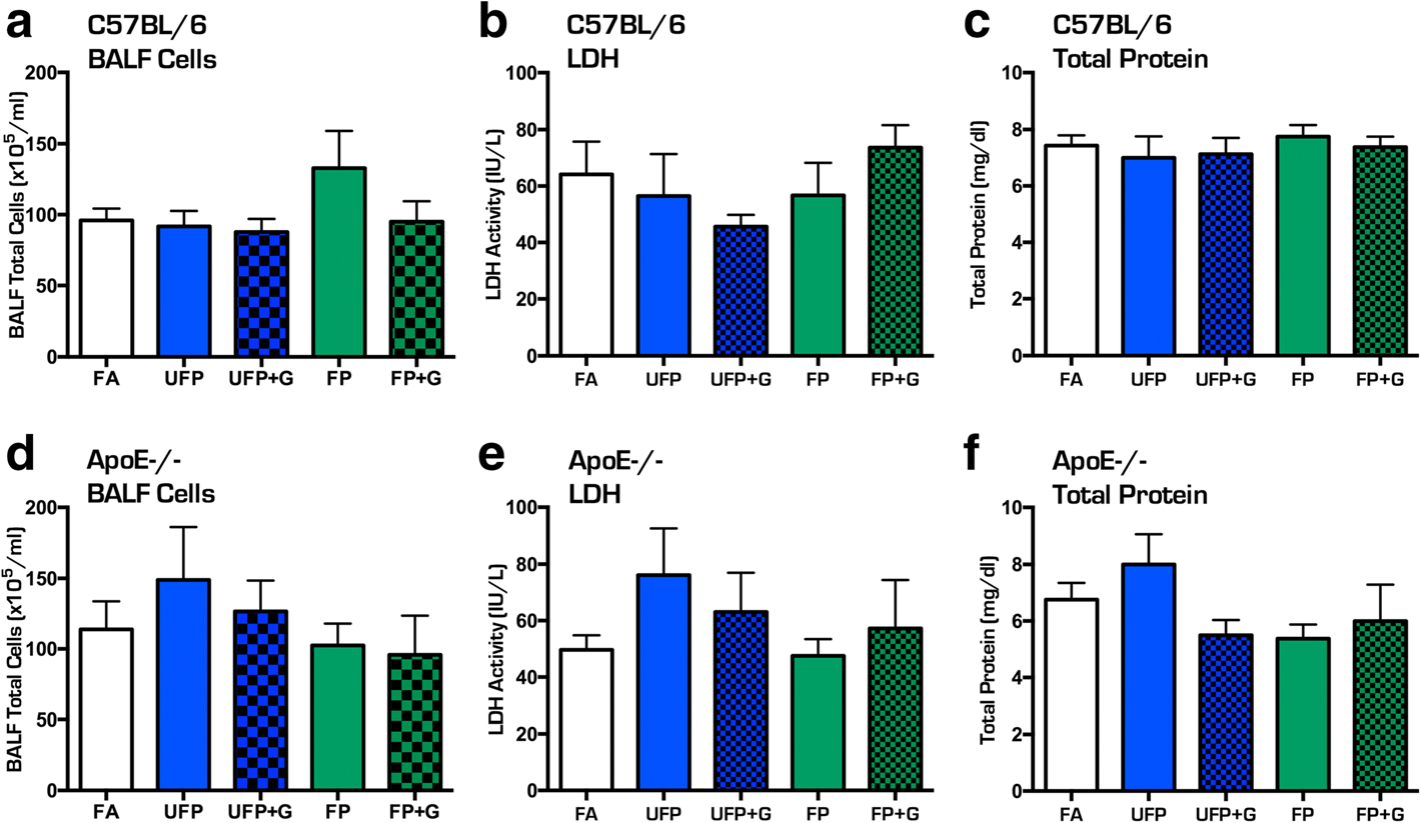
Assessment of bronchial lavage fluid for cellular infiltration after acute (1-day) exposure to atmospheres. Acute exposure to UFP, UFP + G, FP, and FP + G does not induce cellular infiltration into the pulmonary system in the C57BL/6 mice as indicated by no change in bronchial lavage fluid (BALF) assessment of (**a**) total cell counts, (**b**) lactate dehydrogenase (LDH) activity, and (**c**) total protein (albumin). A similar lack of effect was observed in ApoE^−/−^ mice for (**d**) total cell counts, (**e**) lactate dehydrogenase (LDH) activity, and (**g**) total protein (albumin). *n* = 7–8 per group, *p < .05; **p < .01; ***p < .001

**Fig. 4:**
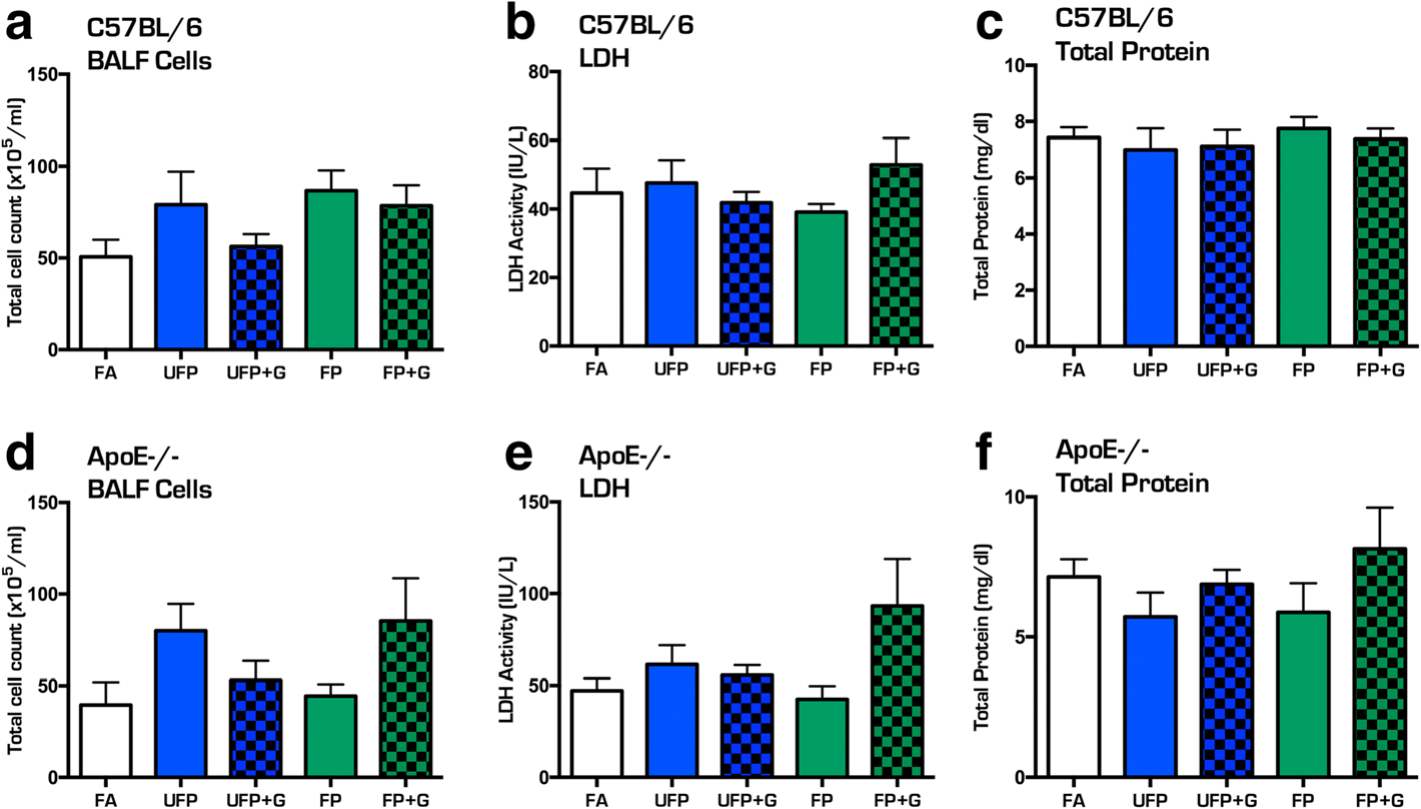
Assessment of bronchial lavage fluid for cellular infiltration after chronic (30-day) exposure to atmospheres. Chronic exposure to UFP, UFP + G, FP, and FP + G does not induce cellular infiltration into the pulmonary system in the C57BL/6 mice as indicated by no changes in bronchial lavage fluid (BALF) assessment of (**a**) total cell counts, (**b**) lactate dehydrogenase (LDH) activity, or (**c**) total protein (albumin). A similar lack of effect was observed in ApoE^−/−^ mice for (**d**) total cell counts, (**e**) lactate dehydrogenase (LDH) activity, and (**g**) total protein (albumin). *n* = 7–8 per group, *p < .05; **p < .01; ***p < .001

Mice in all groups exhibited significant increases in body weight during the course of the chronic 30-day exposure (Fig. 2a, b); however, when normalized to starting body weights, the UFP + G exposed C57BL/6 and ApoE^−/−^ mice exhibited a clear reduction in this overall growth compared to control (Fig. 2c, d). Assessment of BALF for markers of inflammation, including total cell counts, LDH activity, and total protein, indicated no significant response to either acute (Fig. 3a–f) or chronic (Fig. 4a–f) exposure to PM or PM + G in either mouse strain. Additionally, we did not see significant influx of neutrophils, lymphocytes or eosinophils in any group. All one-way ANOVA analyses are provided in Table 2 for bronchial lavage fluid assessments.

**Table 2:** One-way ANOVA statistical analysis of BALF cells, total protein, and LDH

Strain	Exposure	Quantification	*F*-value	*P*-value
C57BL/6	Acute	BALF Cells	F(4,32) = 1.437	ns
C57BL/6	Acute	Total Protein	F(4,33) = 0.7008	ns
C57BL/6	Acute	LDH	F(4,33) = 0.8263	ns
ApoE^−/−^	Acute	BALF Cells	F(4,31) = 0.6264	ns
ApoE^−/−^	Acute	Total Protein	F(4,31) = 2.146	ns (.09)
ApoE^−/−^	Acute	LDH	F(4,31) = 0.9956	ns
C57BL/6	Chronic	BALF Cells	F(4,23) = 1.855	ns
C57BL/6	Chronic	Total Protein	F(4,34) = 0.3106	ns
C57BL/6	Chronic	LDH	F(4,34) = 0.8727	ns
ApoE^−/−^	Chronic	BALF Cells	F(4,22) = 2.058	ns
ApoE^−/−^	Chronic	Total Protein	F(4,32) = 1.057	ns
ApoE^−/−^	Chronic	LDH	F(4,33) = 2.374	ns (.07)

**Fig. 5:**
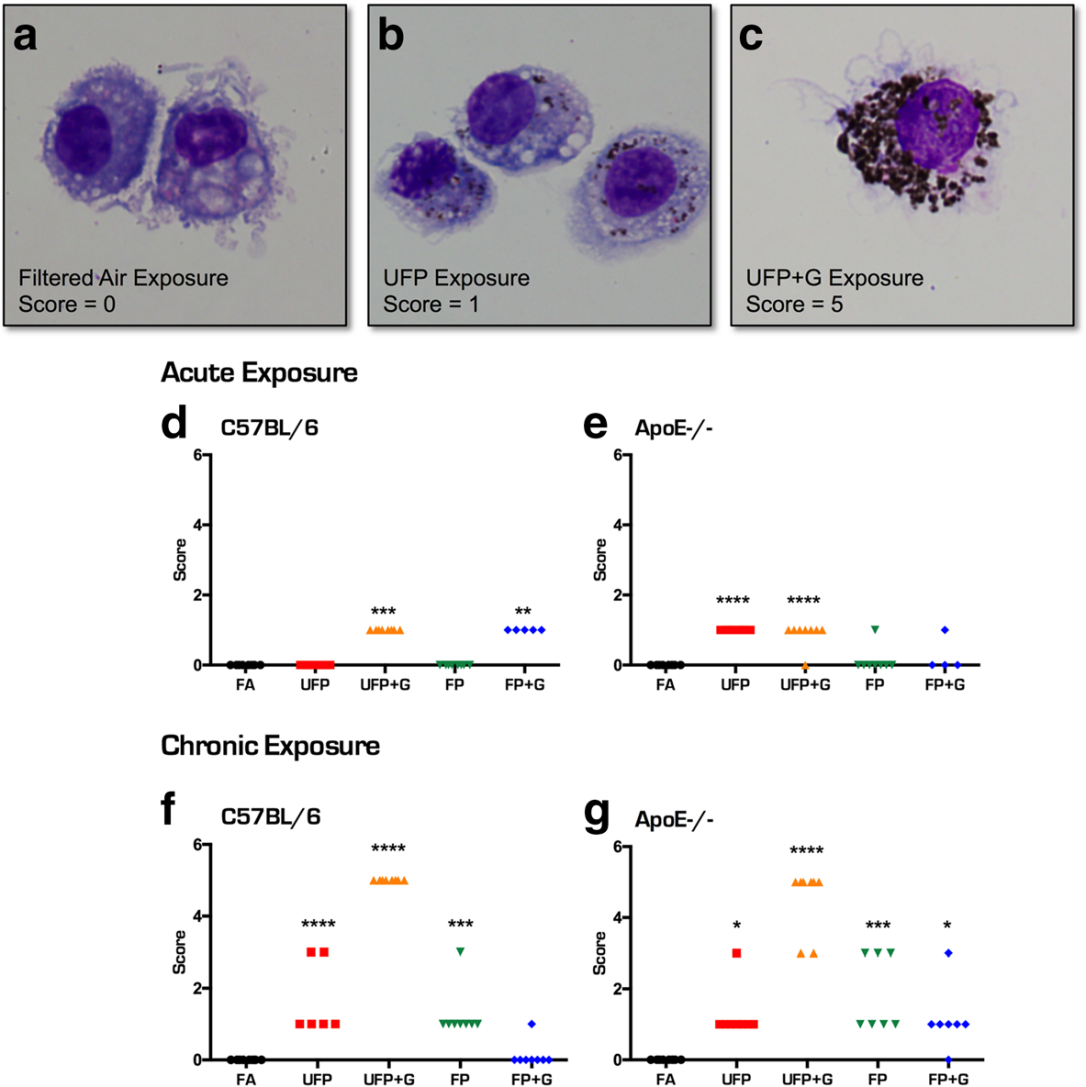
Particulate matter uptake in bronchial macrophages after acute and chronic exposure to atmospheres. Representative images of macrophages in the bronchial lavage fluid for (**a**) animals exposed to filtered air, (**b**) animals exposed to UFP, and (**c**) animals exposed to UFP + G. Images demonstrate the rubric for particulate matter uptake scoring on a scale of 0–5. Acute (1-day) exposure resulted in significant uptake of particulate matter in the (**d**) UFP + G and FP + G exposure groups in C57BL/6 animals and in the (**e**) UFP and UFP + G exposure groups in in ApoE^−/−^ animals. Chronic (30-day) exposure resulted in significant uptake of particulate matter in the (**f**) UFP, UFP + G, and FP exposure groups in C57BL/6 animals, and in (**g**) all exposure groups, UFP, UFP + G, FP, FP + G in ApoE^−/−^ animals. *n* = 7–8 per group, *p < .05; **p < .01; ***p < .001; ****p < .0001

Despite the lack of cellular infiltration into the lungs, macrophages in the BALF did reveal an interesting pattern of PM uptake. Visualization of resident lung macrophages after chronic exposure indicated significant phagocytosis of PM in both strains of mice for all atmospheres, which was qualitatively scored by a blinded operator (Fig. 5a–c). Scoring of PM uptake after acute exposures indicated moderate, but significant accumulation of phagocytized PM in the UFP + G and FP + G groups in C57BL/6 animals (Fig. 5d, *p* < .0001) and significant accumulation of the UFP and UFP + G particles in ApoE^−/−^ animals (Fig. 5e, *p* < .0001). Chronic exposure resulted in more dense accumulation of particulate matter and an apparent potentiation of uptake in both strains of mice exposed to UFP + G. Significant accumulation of UFP (*p* < .0001), UFP + G (*p* < .0001), and FP (*p* < .001) particles occurred in the C57BL/6 animals (Fig. 5f), while ApoE^−/−^ animals had significant accumulation of particles in all chronic exposure groups (Fig. 5g). Table 3 provides all one-way ANOVA values and posthoc analyses for the quantification of accumulation of particulate matter. Thus, chronic co-exposure of gases and UFP led to clearly potentiated phagocytosis of PM in comparison to other exposures, despite identical mass concentrations of PM and no measurable increase in lavagable cells in any group.

**Table 3:** One-way ANOVA statistical analysis of particulate matter uptake in lung

Strain	Exposure	*F*-value	*P*-value	Posthoc analysis
C57BL/6	Acute	Kruskal-Wallis, 36.00	*p* < .0001	UFP + G, FP + G (Dunn’s)
ApoE^−/−^	Acute	Kruskal-Wallis, 25.24	*p* < .0001	UFP, UFP + G (Dunn’s)
C57BL/6	Chronic	F(4,33) = 32.84	*p* < .0001	UFP, UFP + G, FP
ApoE^−/−^	Chronic	F(4,31) = 34.87	*p* < .0001	UFP, UFP + G, FP, FP + G

**Table 4:** One-way ANOVA statistical analysis of protein expression in BALF

Strain	Exposure	Protein	*F*-value	*P*-value	Posthoc analysis
C57BL/6	Chronic	CXCL1	F(4,34) = 45.81	*p* < .0001	UFP + G
C57BL/6	Chronic	IL-1β	F(4,33) = 1.350	ns	none
C57BL/6	Chronic	IL-6	F(4,34) = 1.088	ns	none
C57BL/6	Chronic	TNFα	F(4,34) = 11.32	*p* < .0001	UFP + G
ApoE^−/−^	Chronic	CXCL1	F(4,32) = 12.53	*p* < .0001	UFP + G
ApoE^−/−^	Chronic	IL-1β	F(4,32) = 1.848	ns	none
ApoE^−/−^	Chronic	IL-6	F(4,32) = 1.564	ns	none
ApoE^−/−^	Chronic	TNFα	F(4,32) = 4.864	*p* = .0035	UFP + G

**Table 5:** Two-way ANOVA statistical analysis of CXCL1 protein expression in BALF

Strain	Exposure	Protein	*F*-value	*P*-value	Posthoc analysis
C57BL/6	Chronic	CXCL1	Interaction: F(1,26) = 11.41	*p* = .0023	UFP + G
			Strain: F(1, 26) = 6.534	*p* = 0.0168	
			Exposure: F(1,26) = 84.11	*p* < .0001	

### Cytokine induction in lung lavage

**Fig. 6:**
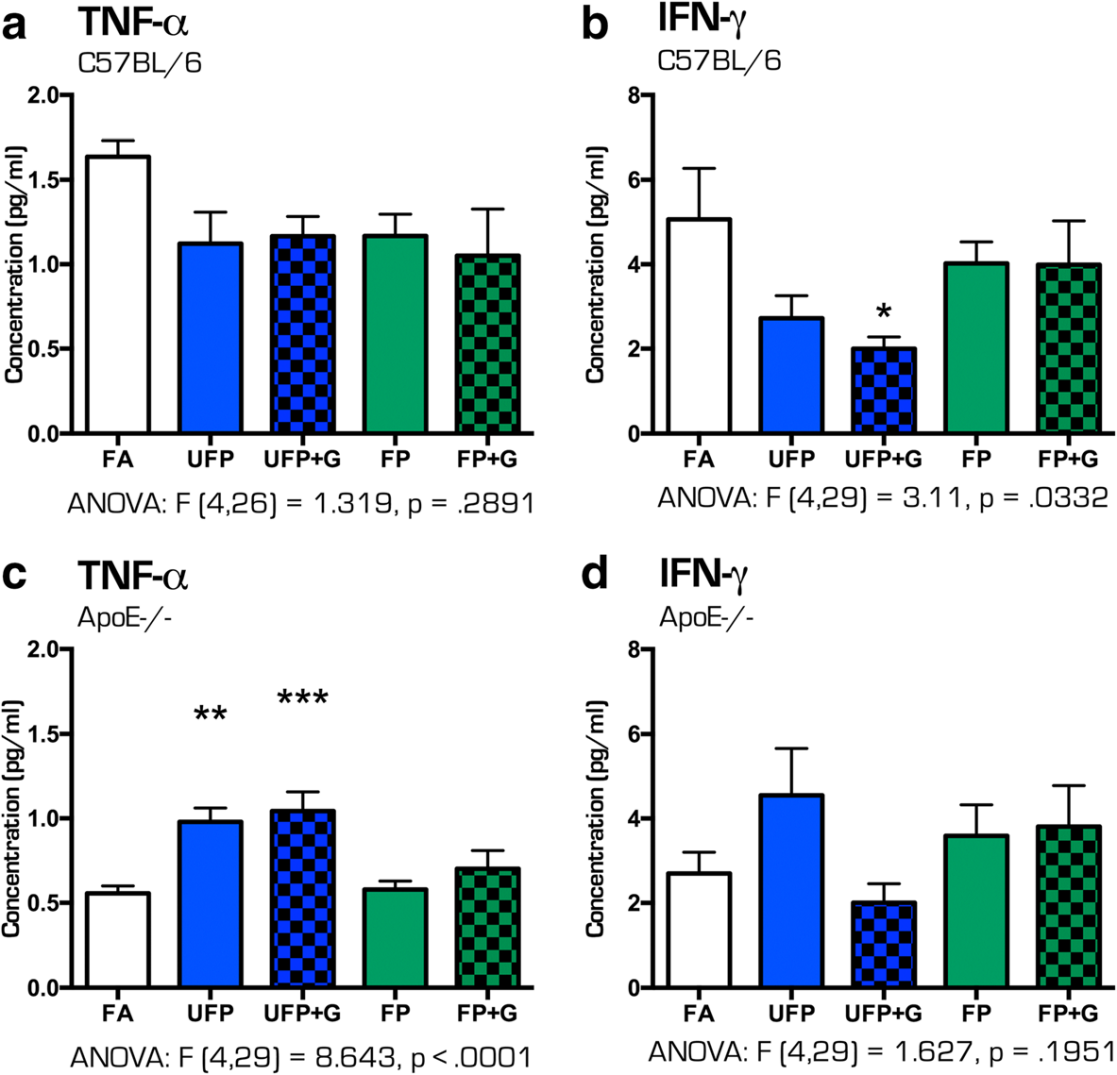
Cytokine protein expression profile in bronchial lavage fluid after acute (1-day) exposure to atmospheres. After 1-day of exposure to the atmospheres, bronchial lavage fluid (BALF) was obtained from animals and assessed for cytokine protein expression for (**a**) TNF-α in C57BL/76 BALF, (**b**) IFN-γ in C57BL/6 BALF, (**c**) TNF-α in ApoE^−/−^ BALF, and (**d**) IFN-γ in ApoE^−/−^ BALF. No change in (**a**) TNF-α expression was observed in C57BL/6 animals, while (**b**) IFN-γ expression was significantly reduced in the UFP + G exposure group in the same animals. In ApoE^−/−^ animals, (**c**) TNF-α protein expression was increased in the UFP and UFP + G groups with (**d**) no significant change in IFN- γ expression. *n* = 7–8 per group, *p < .05; **p < .01; ***p < .001; ****p < .0001

**Fig. 7:**
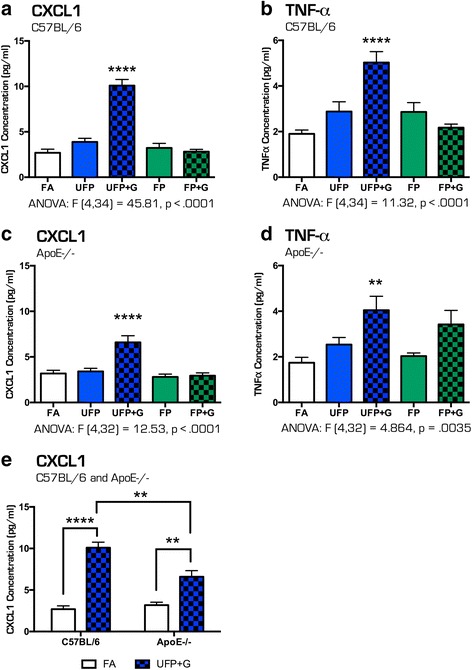
Cytokine protein expression profile in bronchial lavage fluid after chronic (30-day) exposure to atmospheres. After 30-days of exposure to the atmospheres, bronchial lavage fluid (BALF) was obtained from animals and assessed for cytokine protein expression for the (**a**) CXCL1 in C57BL/6 BALF, (**b**) TNF-α in C57BL/6 BALF, (**c**) CXCL1 in ApoE^−/−^ BALF and (**d**) TNF-α in ApoE^−/−^ BALF. The most significant changes were induced by exposure to UFP + G: (**a**) CXCL1 expression in C57BL/6 animals, (**b**) TNF-α expression in C57BL/6 animals, (**c**) CXCL1 expression in ApoE^−/−^ animals, and (**d**) TNF-α expression in ApoE^−/−^ were all significantly increased in after chronic exposure to UFP + G. (**e**) CXCL1 expression was significantly increased in C57BL/6 animals in response to UFP + G compared to ApoE^−/−^ animals. *n* = 7–8 per group, *p < .05; **p < .01; ***p < .001; ****p < .0001

A single exposure to the MVE-derived atmospheres in C57BL/6 mice caused no obvious induction of cytokines in the lung (Fig. 6a, b). Interestingly, interferon-γ (IFN-γ) was significantly reduced in the lavage of UFP + G-exposed C57BL/6 mice (*p* < .05). Conversely, ApoE^−/−^ mice had increased expression of TNFα in the lung lavage after exposure to either UFP (*p* < .01) or UFP + G (*p* < .001) and no change in IFN-γ expression (Fig. 6c, d). Numerous other assayed cytokines were unaltered by acute exposure in either strain (data not shown). After 30 days of exposure, a clear picture emerged with both strains exhibiting a robust and selective increase in CXCL1 and TNFα protein expression only in the UFP + G group, (Fig. 7a–c, *p* < .0001, d, *p* < .001). CXCL1 increased expression was significantly greater in C57BL/6 mice than in ApoE^−/−^ mice (Fig. 7e, *p* < .001), with a significant main effect of exposure (F(1,26) = 84.11, *p* < .0001), main effect of animal strain (F(1,26) = 6.534, *p* < .0168), and a significant interaction between exposure and strain, (F(1, 26) = 11.41, *p* < .0023). Table 4 provides all one-way ANOVA values and posthoc analyses for quantification of cytokine protein expression in the lung lavage after acute and chronic exposures in both strains of mice. Table 5 provides 2-way ANOVA values and posthoc analyses for CXCL1 expression in the lung lavage after chronic exposure to UFP+G in both strains of mice.

### Acute exposure and neuroinflammatory effects in the hippocampus

**Fig. 8:**
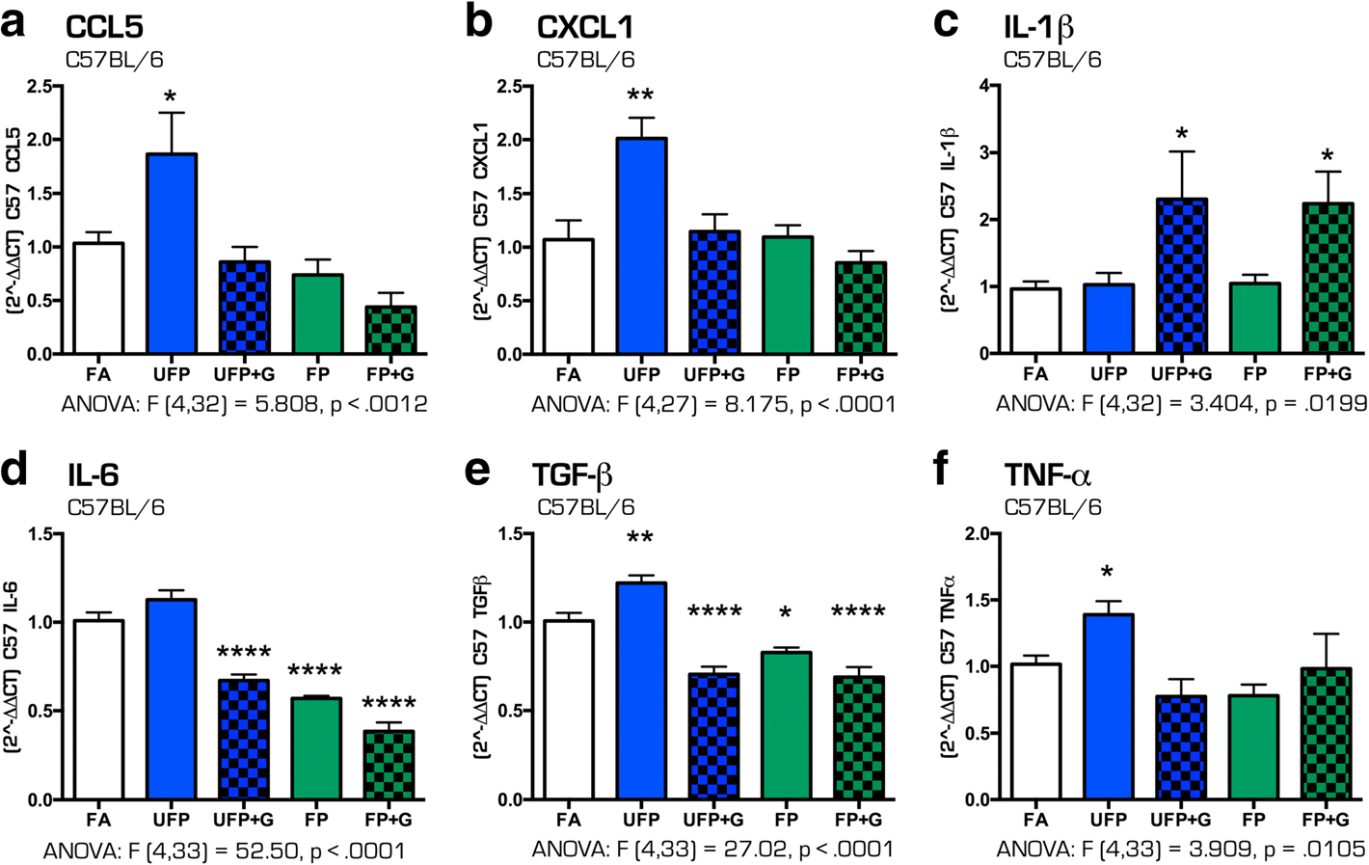
Cytokine expression profile in the C57BL/6 hippocampus after acute (1-day) exposure to atmospheres. After 1-day of exposure to atmospheres, cytokine mRNA expression was assessed in the hippocampus of the C57BL/6 mouse brain for the following: (**a**) Ccl5, (**b**) Cxcl1, (**c**) IL-1β, (**d**) IL-6, (**e**) Tgf-β, and (**f**) Tnf-α. Exposure to UFP significantly altered mRNA expression of (**a**) Ccl5, (**b**) Cxcl1, (**c**) IL-1β, (**e**) Tgf-β, and (**f**) Tnf-α. Exposure to UFP﻿+﻿﻿﻿G or FP+G significantly increased mRNA expression of (**c**) IL-1β. Exposure to UFP + G, FP and FP + G significantly decreased mRNA expression of (**d**) IL-6 and (**e**) Tgf-β. *n* = 7–8 per group, *p < .05; **p < .01; ***p < .001; ****p < .0001

To determine the effect of particulate matter size and composition on the potential for neuroinflammation, quantification of mRNA expression of several cytokines in the hippocampus in response to exposures was performed. Despite minimal impacts on pulmonary outcomes, acute exposure induced significant changes in the expression of several cytokines in the hippocampus of both C57BL/6 and ApoE^−/−^ animals suggesting that particulate matter induces neuroinflammation even after one day of exposure (Fig. 8a–f). In C57BL/6 mice, acute exposure to UFP alone resulted in increased cytokine expression of *Ccl5* (Fig. 8a, *p* < .05), *Cxcl1* (Fig. 8b, *p* < .01), *Tgf-β* (Fig. 8e, *p* < .05), and *Tnf-α* (Fig. 8f, *p* < .05) in the hippocampus. UFP + G and FP + G exposure, however, only increased hippocampal mRNA expression of *Il-1β* (Fig. 8c, *p* < .05), while reducing expression of *Il-6* (Fig. 8d, *p* < .0001). Interestingly, acute exposure to FP and FP + G also resulted in decreased expression of *Il-6* (Fig. 8d, *p* < .0001) and *Tgf-β* (Fig. 8e, *p* < .05 and *p* < .0001, respectively) in the C57BL/6 mice.

**Fig. 9:**
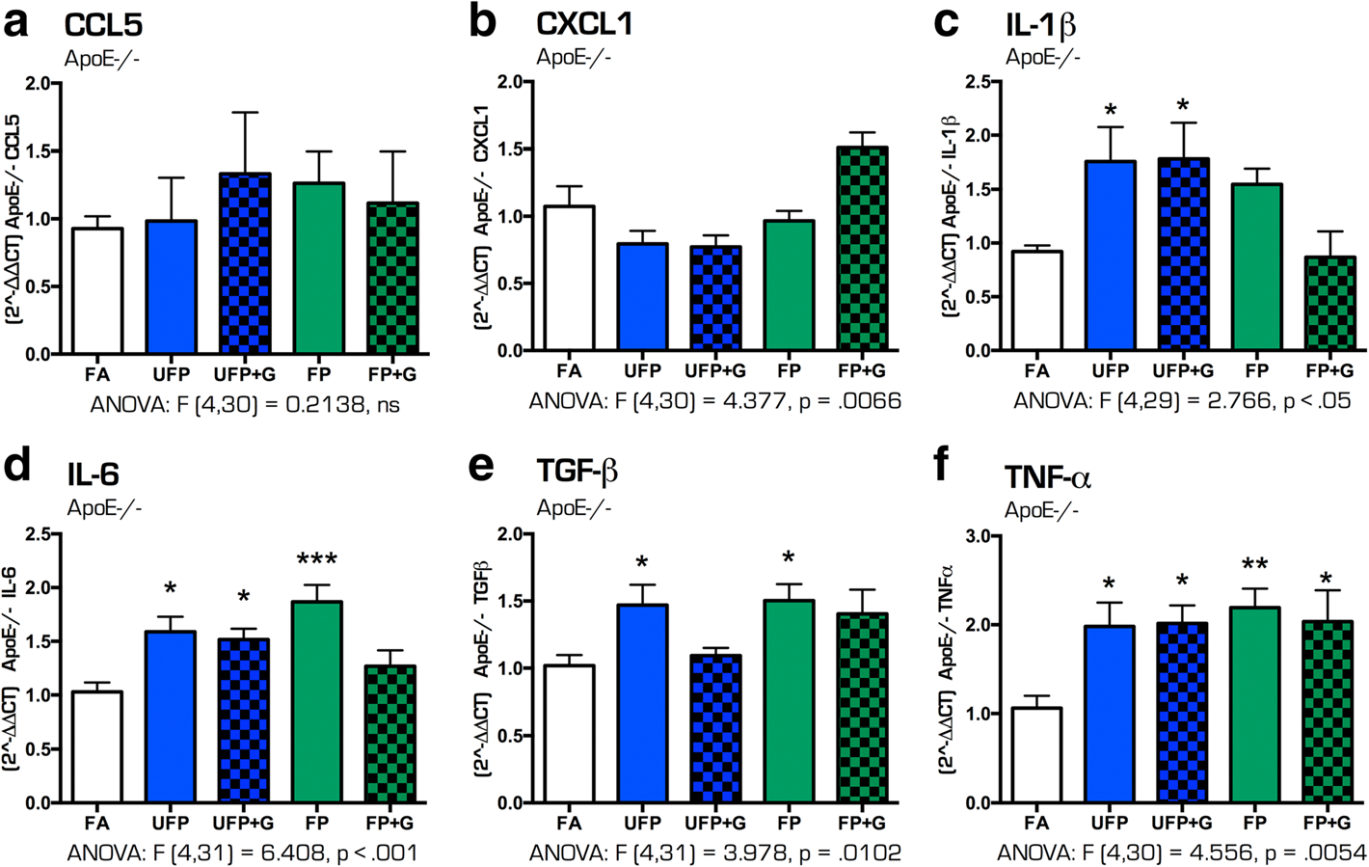
Cytokine expression profile in the ApoE^−/−^ hippocampus after acute (1-day) exposure to atmospheres. After 1-day of exposure to atmospheres, cytokine mRNA expression was assessed in the hippocampus of the ApoE^−/−^ mouse brain for the following: (**a**) Ccl5, (**b**) Cxcl1, (**c**) IL-1β, (**d**) IL-6, (**e**) Tgf-β, and (**f**) Tnf-α. None of the exposures significantly altered mRNA expression of (**a**) Ccl5 or (**b**) Cxcl1. Exposure to UFP and UFP + G significantly altered mRNA expression of (**c**) IL-1β, (**d**) IL-6, (**e**) Tgf-β, and (**f**) Tnf-α, with exposures having the greatest impact on (**f**) Tnf-α expression. *n* = 7–8 per group, *p < .05; **p < .01; ***p < .001

Different results were found for acute exposures in ApoE^−/−^ mice, which appeared to be more prone to broad inflammatory responses to most exposures. *Ccl5* and *Cxcl1* expression did not appear to be increased by any atmosphere after a single exposure (Fig. 9a, b). Acute exposure to UFP alone resulted in significantly increased expression of *Il-1β* (Fig. 9c, *p* < .05), *Il-6* (Fig. 9d, *p* < .05), *Tgf-β* (Fig. 9e, *p* < .05) and *Tnf-α* (Fig. 9e, *p* < .05). While *Il-6* and *Tgf-β* expression were decreased in the hippocampus of C57BL/6 mice for most atmospheres, ApoE^−/−^ mice exhibited opposite trends with *Il-6* elevated in UFP (*p* < .05), UFP + G (*p* < .05) and FP (*p* < .001) groups (with no difference in FP + G, Fig. 9d) and *Tgf-β* elevated in UFP (*p* < .05) and FP (*p* < .05) exposures and unchanged by addition of gases (Fig. 9e). Hippocampal *Tnf-α* was uniformly elevated by all single exposures in ApoE^−/−^ mice, approximately 2-fold (Fig. 9f, *p* < .05).

### Chronic exposure and neuroinflammatory effects in the hippocampus

**Fig. 10:**
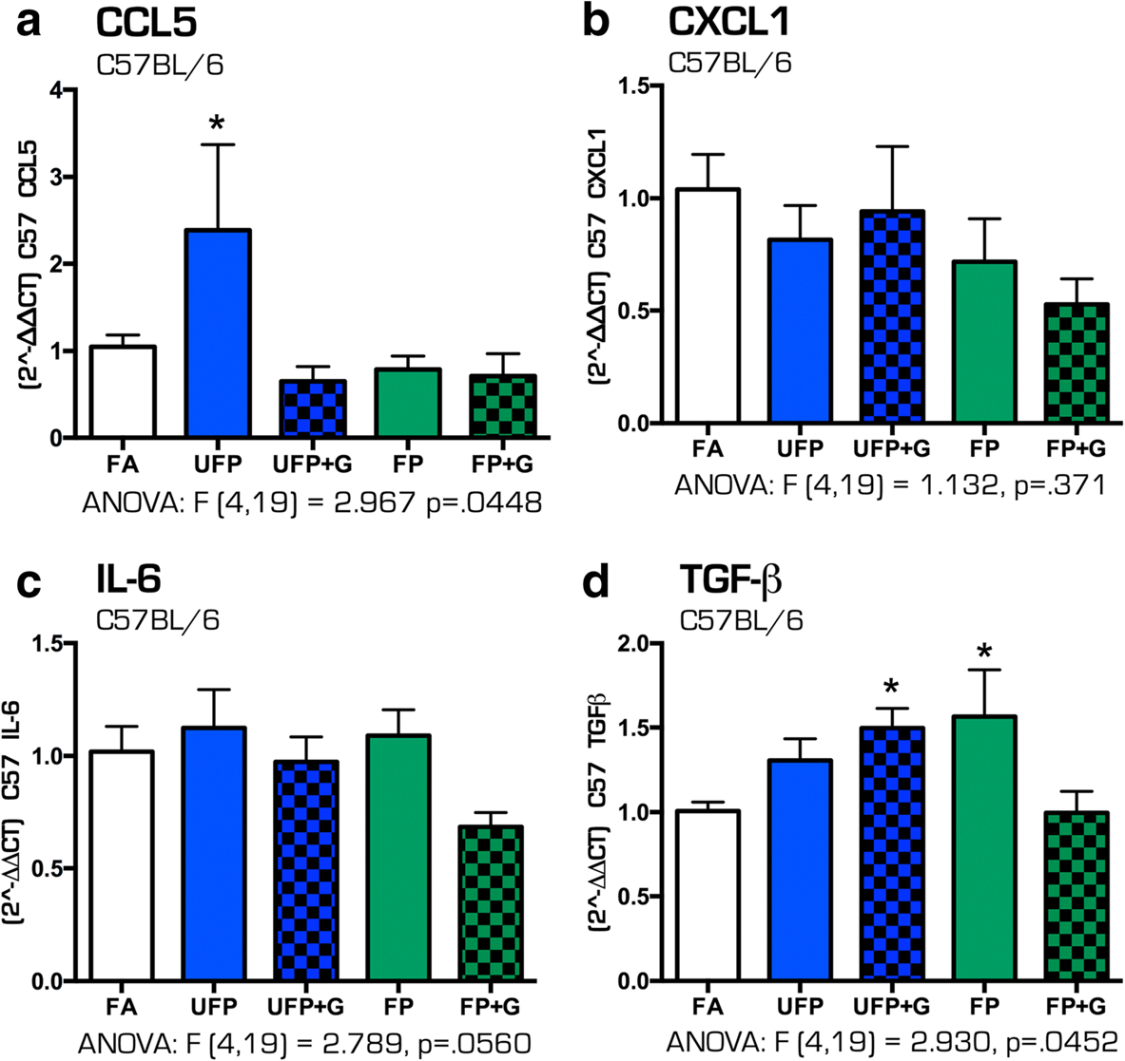
Cytokine expression profile in the C57BL/6 hippocampus after chronic (30-day) exposure to atmospheres. After 30 days of exposure to atmospheres, cytokine mRNA expression was assessed in the hippocampus of the C57BL/6 mouse brain for the following: (**a**) Ccl5, (**b**) Cxcl1, (**c**) IL-6, and (**d**) Tgf-β. UFP exposure only increased (**a**) Ccl5 mRNA expression after thirty days, while UFP + G expression increased (**d**) Tgf-β. *n* = 7–8 per group, *p < .05; **p < .01; ***p < .001

After 30 days of exposure to the four atmospheres, few inflammatory effects were noted in C57BL/6 mice. Chronic exposure to UFP resulted in significantly increased expression of *Ccl5* (Fig. 10a, *p* < .05), with no changes in *Cxcl1* nor *IL-6* cytokine expression (Fig. 10b–c). UFP + G and FP exposures led to significant increased expression of hippocampal *Tgf-β* (Fig. 10d, *p* < .05). No other inflammatory gene changes, including *Ccl1* and *Et1* (data not shown), were observed in hippocampal tissues from the WT mice.

**Fig. 11:**
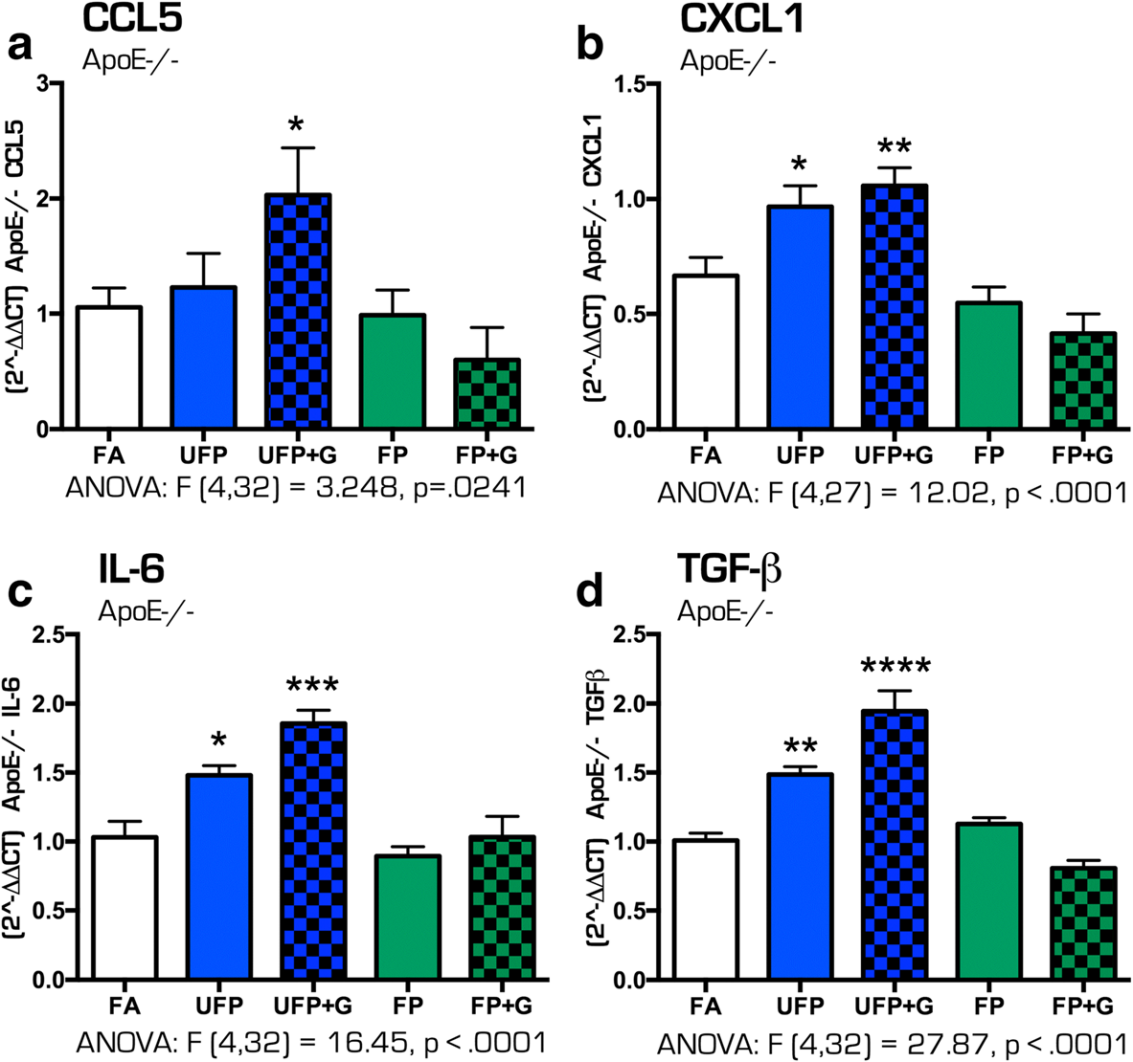
Cytokine expression profile in the ApoE^−/−^ hippocampus after chronic (30-day) exposure to atmospheres. After 30 days of exposure to atmospheres, cytokine mRNA expression was assessed in the hippocampus of the ApoE^−/−^ mouse brain for the following: (**a**) Ccl5, (**b**) Cxcl1, (**c**) IL-6, and (**d**) Tgf-β. UFP exposure increased (**b**) Cxcl1, (**c**) IL-6, and (**d**) Tgf-β mRNA expression after thirty days, while UFP + G expression increased expression of all cytokines assessed after chronic exposure (**a**) Ccl5, (**b**) Cxcl1, (**c**) IL-6, and (**d**) Tgf-β. *n* = 7–8 per group, *p < .05; **p < .01; ***p < .001; ****p < .0001

In contrast, the 30-day chronic exposure paradigm induced a greater and more consistent pattern of inflammatory responses in the hippocampus of ApoE^−/−^ animals. Assessment of the same cytokine profiles revealed significantly increased expression of *Ccl5* (Fig. 11a, *p* < .05), *Cxcl1* (Fig. 11b, *p* < .05, *p* < .01), *Il-6* (Fig. 11c, *p* < .05, *p* < .001) and *Tgf-β* (Fig. 11d, *p* < .01, *p* < .0001), with the greatest changes in cytokine expression resulting from the UFP + G exposure atmospheres, and a smaller but notable increase from exposure to UFP alone. Similar to C57BL/6 animals, no change in mRNA expression of *Ccl2* was determined (data not shown). Table 6 includes all one-way ANOVA data values for the quantification of mRNA expression for all cytokines measured in the hippocampus of C57BL/6 and ApoE^−/−^ mice after acute and chronic exposures. These results suggest that C57BL/6 mice exhibit healthy adaptive responses to minimize neuroinflammatory effects of vehicle-derived pollutant exposure, while the hypercholesterolemic mice are impaired in such homeostatic compensatory mechanisms, leading to measureable neuroinflammation.

**Table 6:** One-way ANOVA statistical analysis of qPCR gene expression in hippocampus

Strain	Exposure	Gene	*F*-value	*P*-value	Posthoc analysis relative to control
C57BL/6	Acute	Ccl5	F(4,32) = 5.808	*p* < .0012	UFP
C57BL/6	Acute	Cxcl1	F(4,27) = 8.175	*p* < .0001	UFP
C57BL/6	Acute	IL-1β	F(4,32) = 3.404	*p* = 0.0199	UFP + G, FP + G
C57BL/6	Acute	IL-6	F(4,33) = 52.50	*p* < .0001	UFP + G, FP, FP + G (decreased)
C57BL/6	Acute	Tgf-β	F(4,33) = 27.02	p < .0001	UFP (increased), UFP + G, FP, FP + G (decreased)
C57BL/6	Acute	Tnf-α	F(4,33) = 3.909	*p* = .0105	UFP
C57BL/6	Acute	Vcam-1	F(4,32) = 1.205	ns	none
ApoE^−/−^	Acute	Ccl5	F(4,30) = 0.2138	ns	none
ApoE^−/−^	Acute	Cxcl1	F(4,30) = 4.377	*p* = 0.0066	none
ApoE^−/−^	Acute	IL-1β	F(4,29) = 2.766	*p* = 0.05	UFP, UFP + G
ApoE^−/−^	Acute	IL-6	F(4,31) = 6.408	*p* < 0.001	UFP, UFP + G, FP
ApoE^−/−^	Acute	Tgf-β	F(4,31) = 3.978	*p* = .0102	UFP, FP
ApoE^−/−^	Acute	Tnf-α	F(4,30) = 4.556	*p* = .0054	UFP, UFP + G, FP, FP + G
ApoE^−/−^	Acute	Vcam-1	F(4,30) = 8.388	*p* < .0001	FP
C57BL/6	Chronic	Ccl5	F(4,19) = 2.967	*p* = .0448	UFP
C57BL/6	Chronic	Ccl2	F(4,18) = 1.398	ns	none
C57BL/6	Chronic	Cxcl1	F(4,19) = 1.132	ns	none
C57BL/6	Chronic	Et1	F(4,19) = 1.866	ns	none
C57BL/6	Chronic	IL-6	F(4,19) = 2.789	ns (*p* = .0560)	none
C57BL/6	Chronic	Tgf-β	F(4,19) = 2.930	*p* = .0452	UFP + G, FP
C57BL/6	Chronic	Vcam-1	F(4,19) = 2.962	*p* = .0465	UFP + G, FP
ApoE^−/−^	Chronic	Ccl5	F(4,32) = 3.248	*p* = .0241	UFP + G
ApoE^−/−^	Chronic	Ccl2	F(4,29) = 1.422	ns	none
ApoE^−/−^	Chronic	Cxcl1	F(4,27) = 12.02	*p* < .0001	UFP, UFP + G
ApoE^−/−^	Chronic	Et1	F(4,32) = 1.78	ns	none
ApoE^−/−^	Chronic	IL-6	F(4,32) = 16.45	*p* < .0001	UFP, UFP + G
ApoE^−/−^	Chronic	Tgf-β	F(4,32) = 27.87	*p* < .0001	UFP, UFP + G
ApoE^−/−^	Chronic	Vcam-1	F(4,29) = 3.416	*p* = .0209	none

### Chronic exposure to MVE and MVE + gases alters expression of genes involved in cerebral vasculature

**Fig. 12:**
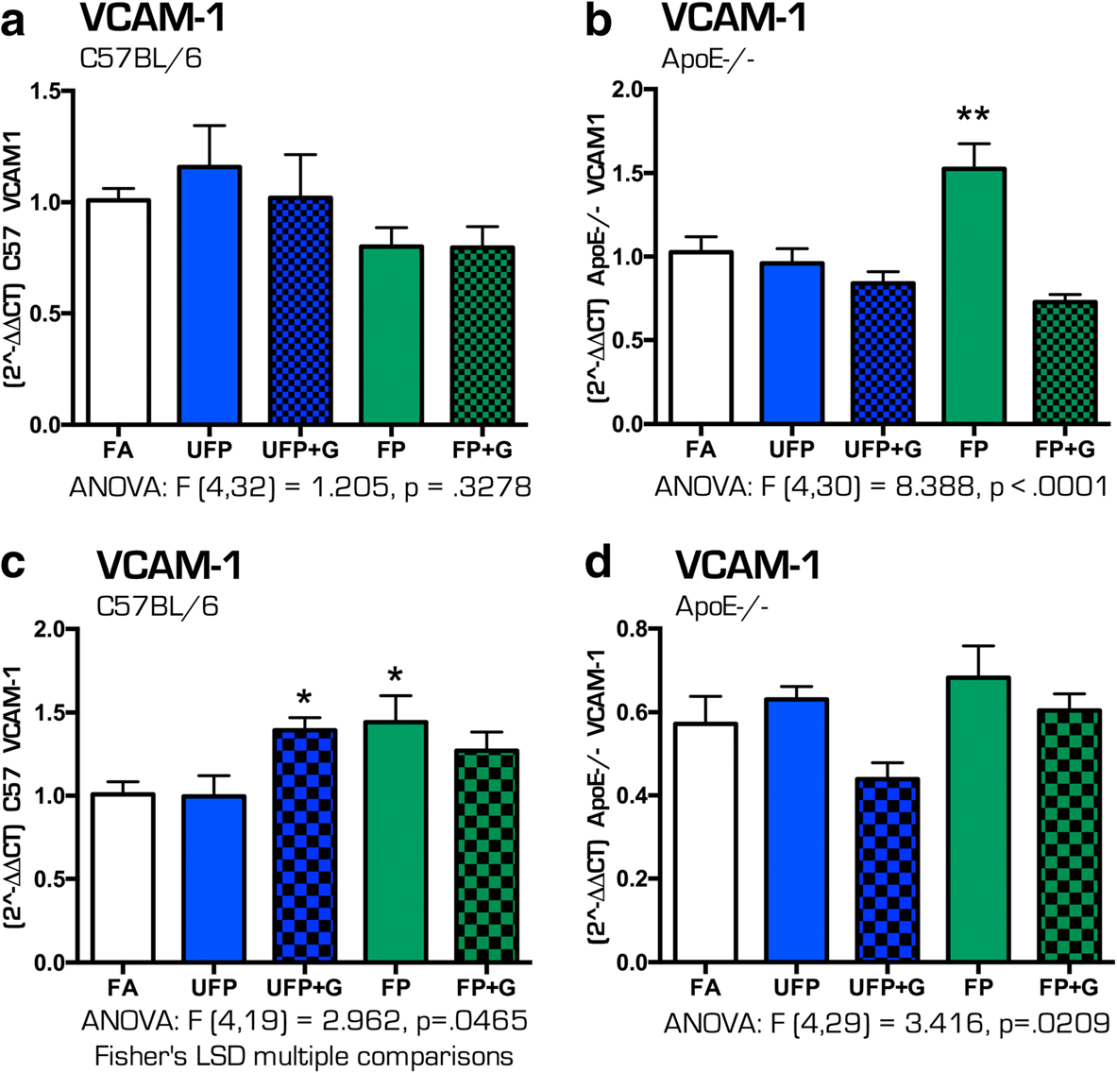
Vasculature mRNA expression in the hippocampus of C57BL/6 and ApoE^−/−^ mice after acute and chronic exposure to atmospheres. Expression of Vcam-1 mRNA was determined after acute exposures in the (**a**) hippocampus of C57BL/6 mice and (**b**) hippocampus of ApoE^−/−^ mice. The only significant effect was that of acute exposure to FP on Vcam-1 in the ApoE^−/−^ hippocampus. Expression of Vcam-1 mRNA was determined after chronic exposures in the (**c**) hippocampus of C57BL/6 mice and (**d**) hippocampus of ApoE^−/−^ mice. UFP + G and FP significantly increased Vcam-1 expression in the C57BL/6 hippocampus after 30 days of exposure. *n* = 7–8 per group, *p < .05; **p < .01; ***p < .001

To determine if the size and composition of particulate matter impacted the expression of vascular inflammatory response genes in the brain, mRNA expression of *Vcam1* and *Et1* was assessed. Acute exposures to vehicle-derived pollutants did not alter the expression of *Vcam1* in the hippocampus of C57BL/6 mice (Fig. 12a). In ApoE^−/−^ mice, acute exposure to FP only caused a 50% increase in *Vcam1* (Fig. 12b, *p* < .0001). Chronic exposure to UFP + G and FP increased *Vcam1* expression in C57BL/6 mice (Fig. 12c, *p* < .05) but had no effect on *Vcam1* expression in the hippocampus of ApoE^−/−^ mice (Fig. 12d). No effect of chronic exposure was determined on the mRNA expression of *Et1* in either strain of animal (Additional file 1: Figure S1). See Table 6 for all ANOVA and F values for *Vcam1* and *Et1* expression after acute and chronic exposure in both animal strains.

## Discussion

Recent epidemiological studies have linked traffic-related air pollution to adverse neurological outcomes [2, 30] with several toxicological reports demonstrating that PM exposure has the capacity to alter neural development [31, 32], induce neuroinflammation [33], impair cognition [34], and potentially induce neurodegeneration [35]. However, there is still much debate regarding such findings owing to inconsistent results and a lack of understanding as to how inhalation exposures lead to neurological deficits. The present study adds substantially to the biological plausibility of this phenomenon by providing evidence that neuroinflammatory outcomes of exposure, as assessed via cytokine production, are most pronounced with smaller PM, particularly in the presence of vehicle engine co-pollutants, a likely consequence of a high PM surface area for semivolatile compound adsorption. Furthermore, ApoE^−/−^ mice exhibited clear vulnerability to long-term exposure to ultrafine PM recombined with gases (UFP + G exposure group), suggesting impairment of homeostatic compensatory mechanisms fully intact in C57BL/6 mice with potential neuroprotective function. Which specific aspects of the ApoE^−/−^ mouse model on a high fat diet that contributed to this neuroinflammatory vulnerability remain uncertain. Lastly, data presented here provide an intriguing link to pulmonary effects, with clearly enhanced PM phagocytosis by alveolar macrophages and induction of proinflammatory cytokines in the airways without any indication of neutrophil influx or lung injury. Thus, this study suggests a potential crosstalk between pulmonary and neurological systems in response to inhaled toxicants with clear demonstration of exacerbated toxicological effects when PM is combined with gaseous co-pollutants.

Ample evidence supports a unique and enhanced toxicity from UFP compared to larger PM [36]. Vehicle-derived UFP are generated in the presence of gaseous co-pollutants, including carbon monoxide, oxides of nitrogen, and semivolatile and volatile hydrocarbons [25, 26, 37]. Numerous epidemiological findings highlight a role for roadway proximity or vehicle pollution-specific components as potent drivers of health effects [38, 39], strongly suggesting that freshly-generated PM may have enhanced toxicity. We recently found that, indeed, combined gas and PM mixtures drove more profound vascular toxicity in ApoE^−/−^ mice [23]. As the role of PM surface interactions with gaseous species remained uncertain, we developed the present exposure paradigm to compare different sizes of PM of the same chemical composition in a head-to-head manner. While our findings suggest that certain biological outcomes can be induced by all respirable PM, the most consistent and severe pulmonary effects and neuroinflammatory consequences were clearly manifested in the UFP + G group, consistent with the hypothesis that co-pollutant gases adsorb onto the surface of PM, and greater PM surface area permits enhanced adsorption, leading to more deleterious outcomes. Indeed, the size of UFPs results in deeper deposition in the lung after inhalation and subsequent location within and beyond the lung epithelial barrier with potential uptake into cells resulting in greater potential toxicity [40].

Among the more interesting outcomes from this novel study design, we observed dramatic differences in macrophage phagocytosis of PM in the UFP + G chronic (30 day) exposure group, concurrent with increased TNFα and CXCL1 protein expression in the lavage but not increased cellularity or total protein. Severe accumulation of PM did not occur after a single day of exposure; however, UFP + G exposure led to as much PM uptake as any of the other exposures. Macrophage activation resulting from diesel PM uptake has been shown to cause release of highly toxic factors that activate endothelial cells more so than PM exposure can induce directly, though these factors have yet to be characterized [41]. Mutlu and colleagues demonstrated a role for macrophage-derived cytokines, specifically IL-6, in driving systemic platelet activation [42]. Thus, such phagocytosis and macrophage activation appears mechanistically linked to systemic outcomes. Why the combination of UFP and co-pollutant gases leads to greater phagocytosis that the same amount of denuded UFP or same mass concentration of FP with gases is unclear. However, freshly generated elemental carbon-based PM has been shown to have greater potential to induced oxidative stress in macrophages *in vitro* [43]; this study further indicated that total surface area of different PM samples (all largely comprised of elemental carbon) was a key driver of all oxidative outcomes. A major challenge for understanding the mechanistic basis of the enhanced phagocytosis relates to the need for freshly-generated UFP from the MVE system. Collection of PM from fresh exhaust for later exposure leads to the loss of volatile components that may well be the causative factors. Further study of this phenomenon will, however, provide important information regarding the potency of near-roadway pollution and the pathogenesis of PM-induced systemic disease.

In addition to pulmonary effects, we found the most severe neuroinflammatory consequences were induced by the UFP + G exposure group after chronic exposure in the ApoE^−/−^ animals. Air pollution is correlated with adverse neurodevelopmental outcomes, for which epidemiological evidence suggests a link to autism [44, 45], and neurodegeneration [46] with a potential neuroinflammatory and oxidative stress mechanism of action [13]. The hippocampus, a region of the brain important for learning and memory, is particularly sensitive to environmental toxin exposure leading to disruption of function, neuronal loss, and cognitive decline [47]; therefore, we chose to focus the cytokine profile in this region. While C57BL/6 mice had increased proinflammatory cytokine expression after the acute exposure paradigm, these effects were abolished after chronic exposure. Interestingly, decreased expression of *Il-6* and *Tgf-β* after acute exposures in the hippocampus of C57BL/6 mice was observed. As the balance between pro- and anti-inflammatory processes in the brain is highly dynamic and temporally regulated, especially with IL-6 [48], compensatory mechanisms were potentially already underway 24 h post-acute exposure. TGF-β has been shown to be neuroprotective and reduce brain injury in response to ischemia [49]; thus, lack of TGF-β signaling could indicate that the exposure groups were altering the balance between the pro- and anti-inflammatory cascades in the C57BL/6 hippocampus. After chronic exposure, increased *Tgf-β* expression in the C57BL/6 animals in response to UFP + G may indicate anti-inflammatory protective response, perhaps derived from microglia, to control the pro-inflammatory cascade that occurs earlier in the exposure paradigm. Thus, C57BL/6 mice exhibit healthy adaptive responses to minimize neuroinflammatory effects of vehicle-derived pollutant exposure. In sharp contrast to the C57BL/6 animals, the hypercholesterolemic ApoE^−/−^ mice displayed measurable neuroinflammation via increased cytokine expression, suggesting impaired homeostatic compensatory mechanisms after 30 days of exposure. Anti-inflammatory responses including increased *Cxcl1* and *Tgf-β* expression, potentially from microglia, in the hippocampus may have been combating increased proinflammatory cytokine expression of *Il-6* and *Ccl5*, though IL-6 for its part has been implicated in both pro and anti-inflammatory responses in the brain and may be paramount for healthy aging of the brain [50]. It should be noted that “activation” of microglia, while not directly measured in this study, results in the production of a number of pro- and anti-inflammatory cytokines depending on the environmental niche [51]. Microglia can have divergent responses depending on their localization, hence increased pro-inflammatory and anti-inflammatory cytokines, like IL-6 and TGF-β, in the same temporal range, as in the hippocampus of ApoE^−/−^ mice after chronic exposure. While we cannot definitively state that this cytokine profile is directly from microglia, it is likely that neuroinflammation is occurring after chronic exposure in the ApoE^−/−^ mice, and this could underlie altered microglia activity eventually culminating in neuronal death and neurodegeneration. These effects will be determined in future studies. For now, we can conclude that as observed after acute exposure in the C57BL/6 animals, in ApoE^−/−^ animals, the greatest response in the cytokine profile was elicited by the UFP and UFP + G groups, even after chronic exposure, suggesting that particulate matter size and the recombination of smaller particulate matter with gases induces a greater inflammatory response than larger particles (even when larger particles are combined with gases).

The small, highly complex exposure groups generated for this study appropriately model the type of near-roadway air pollution to which many people are exposed. While several studies have assessed the neuroinflammatory potential of exposure to PM, these studies have focused on larger sized particles, PM_2.5_ and PM_10_, without assessment of particulate matter recombined with gases for modeling typical exposure paradigms in humans. For example, long-term, chronic exposure to PM_2.5_ increases proinflammatory cytokine expression of TNF-α and IL-1β in the rat hippocampus that is concurrent with decreased dendritic arborization, deficits in learning and memory, and depressive-like behaviors [34]. Studies assessing UFP exposure have shown various outcomes including oxidative stress, IL-1α and TNF- α expression in the brain [52], altered MAPK signaling, and elevated GFAP indicative of astrocyte activation in ApoE^−/−^ mice [53]. Early postnatal exposure to UFP results in differential microglial responses dependent on sex and region of the brain, with some areas, such as the hippocampus, responding to UFP via increased microglial activation [31, 54], suggesting enhanced vulnerability to UFP exposure during neurodevelopment. UFP exposure exacerbates natural aging mechanisms in the brain via translocation to brain regions resulting in chronic neuroinflammation concurrent with neurodegenerative outcomes [55–57]. While further characterization of the specific microglial response, the potential for neurodegeneration, and the mechanism by which the exposures induce neuroinflammation (potentially via translocation of the particles) is required, this study is the first to demonstrate neuroinflammatory outcomes using exposure models that include recombination with gases to most accurately model traffic-related air pollution. Furthermore, ApoE^−/−^ mice fed high fat chow were used to model individuals with increased cardiovascular disease (CVD) risk; our results suggest that common traffic emission exposures, whether acute or chronic, could result in more profound neuroinflammation in at-risk populations, such as those with CVD, who may lack the appropriate compensatory mechanisms to combat pro-inflammatory cascades in the brain. Based on these data, we can speculate that long-term exposure to common traffic emissions (UFP + G) would likely result in even greater exacerbation of neuroinflammation, potentially resulting in behavioral manifestations and neurodegeneration, possibly modeling neurological deficits observed in humans.

## Conclusion

We developed an exposure model to determine the contribution of particle size and particle complexity to pulmonary and potential neuroinflammatory outcomes after acute and chronic exposures in C57BL/6 and ApoE^−/−^ animals. Our results showed that chronic exposure to ultrafine carbonaceous particles mixed with vehicular emissions exerted greater effects than UFP alone, or fine particles mixed with the gas phase mixture, suggesting that the surface area of particulates and the interaction of surface-adhering gaseous components enhances pulmonary and systemic toxicity. This outcome implicates freshly generated UFP as inherently more toxic than PM that has aged and lost surface-adhered volatile gases, and further suggests that near-roadway PM may have greater health effects than would be predicted from concentration alone. Future analysis into the potential pathogenesis of neuroinflammatory outcomes by inhaled pollutants and the role of cardiovascular disease as a contributing factor will help resolve important mechanistic questions not addressed by the present study.

## Additional file

Additional file 1: Figure S1. Et-1 mRNA expression in the hippocampus of C57BL/6 and ApoE^−/−^ mice after acute and chronic exposure to atmospheres. Expression of Et-1 mRNA was determined after chronic exposures in the (a) hippocampus of C57BL/6 mice and (b) hippocampus of ApoE^−/−^ mice, with no significant effects induced by any atmospheres. (TIFF 754 kb)
